# Substituting facial movements in singers changes the sounds of musical intervals

**DOI:** 10.1038/s41598-021-01797-z

**Published:** 2021-11-17

**Authors:** Bruno Laeng, Sarjo Kuyateh, Tejaswinee Kelkar

**Affiliations:** 1 RITMO Centre for Interdisciplinary Studies in Rhythm, Time and Motion, University of Oslo, Forskningsveien 3A, 1094 Blindern, 0317 Oslo, Norway; 2 Department of Psychology, University of Oslo, Oslo, Norway; 3 Department of Musicology, University of Oslo, Oslo, Norway

**Keywords:** Neuroscience, Psychology

## Abstract

Cross-modal integration is ubiquitous within perception and, in humans, the McGurk effect demonstrates that seeing a person articulating speech can change what we hear into a new auditory percept. It remains unclear whether cross-modal integration of sight and sound generalizes to other visible vocal articulations like those made by singers. We surmise that perceptual integrative effects should involve music deeply, since there is ample indeterminacy and variability in its auditory signals. We show that switching videos of sung musical intervals changes systematically the estimated distance between two notes of a musical interval so that pairing the video of a smaller sung interval to a relatively larger auditory led to compression effects on rated intervals, whereas the reverse led to a stretching effect. In addition, after seeing a visually switched video of an equally-tempered sung interval and then hearing the same interval played on the piano, the two intervals were judged often different though they differed only in instrument. These findings reveal spontaneous, cross-modal, integration of vocal sounds and clearly indicate that strong integration of sound and sight can occur beyond the articulations of natural speech.

## Introduction

Listening to a song while looking at the face of a singer is a common experience. Intuitively, the musical sounds one hears should depend solely on the sound waves carrying the musical sound while the facial movements of a singer may add expression to the musical performance but remain separate information that is irrelevant to how the notes sound. Yet, a well-known crossmodal bias like the McGurk effect^1^ suggests that at least for the sounds of language, this intuition is incorrect. In fact, pairing the facial movements belonging to one phoneme, while hearing another, generates the perception of another (in-between) phoneme that was in neither the auditory nor the visual signals. In the classic example: /b/ over a visually articulated /g/ yields a fused percept of /d/.

If sight can change what we hear beyond speech, this would indicate that the brain generally integrates the visual input with the auditory input pertaining to a same source or event. According to Bayesian integration theory^2–4^, such a strategy can cope in a statistically optimal fashion with the indeterminacy and variability of information of any sensory channel. In most cases, cross-modal fusion enhances perceptual reliability and the coherence of multisensory representations as a single, unitary, physical event^5–9^.

We surmise that perceptual integrative effects should involve music deeply, since there is ample indeterminacy and variability in its auditory signals. Listeners tend to attribute a wide range of auditory configurations of pitch to a same musical interval and even professional musicians can judge as ‘excellent’ musical tones that can vary up to ± 50 cents or a semitone when listening to instruments with adjustable tuning^10^. When listening to the best singers, experts can judge melodic intervals that are 25 cents off as in-tune^11^. In some styles like opera, intonational variation can even exceed a semitone and go unnoticed by experts^12^. Some have invoked a ‘vocal generosity effect’ where listeners are more likely to call ‘in-tune’ the same sequence of tone intervals when sung than when played identically on a violin^13^.

According to the motor theory of perception^14,15^, one does not identify ‘sound patterns’ but rather ‘sound gestures’. Hence, we expect that facial motion linked to intonation and vocal emission can alter how one hears the sung sounds in a predictable way. Yet, if we perceive spoken words by identifying the vocal tract’s gestures that shape them, then it is likely we perceive the tones’ pitch relations of musical intervals based on visual cues as well, revealing—when these are incongruent—an audiovisual (AV) integration effect characterized by fused tonal percepts.

A few studies have shown integrative effects of visual information on the perception of musical sounds. Switching videos of string players either plucking or bowing can generate a shift, albeit weak, of the perceived timbre of the sounds^16^. The perceived tone duration on a marimba can appear subjectively longer when paired to a long versus short arm gesture^17^ and watching body movements modifies the expressive intentions of a performer^18–20^ and the quality of musical performances^21^, sufficiently to identify from muted videos the winners of real music contests^22^. Most relevantly, two previous studies found evidence that mismatched audiovisual sung intervals affected judgments of tone interval size^23,24^.

Here, we present systematic and strong evidence that spontaneous facial gestures during singing alter the heard interval’s relations, leading to gross deviations in interval size estimates (in Experiment 1) or in failed detections of repeated identical auditory intervals (in Experiment 2). Tone intervals are the minimal structure of melodies, as phonemes are for words. It is known that the extent of head and facial (eyebrows and lips) movements in singers is correlated to the size of the sung intervals or to vocal pitch height^25^. Moreover, observers’ estimates of the size of sung intervals seen in muted videos are correlated to the actual interval size^26^.

Thus, we used different pairs of sung tones forming diatonic musical intervals (e.g., a third, a fifth, an octave, etc.), sometimes congruent (i.e., natural) or incongruent (i.e., cross-dubbed) to the facial gesture. In the first experiment, two groups of individuals estimated what they heard when viewing short videos of one of two singers vocalizing ten different musical intervals. In the incongruent trials of this first experiment, when viewing the facial articulation of a ‘larger’ musical interval but hearing a ‘smaller’ interval, we expected a stretching or ‘expansion’ of the heard interval, reflected in interval size estimates, whereas we expected the reverse arrangement to yield a ‘compression’ in the perception of the interval. Importantly, the degree of alteration of the interval was expected to be proportional to the distance between the audio and the video, with the perfectly congruent match corresponding to no change in estimates (i.e., a zero discrepancy between the heard interval and the distance rating). Moreover, in a second experiment, when comparing piano intervals to the same sung intervals in incongruent videos, we expected individuals to mishear the intervals as ‘different’ when they were acoustically the same and differed only in instrument (voice or piano). Such findings would strongly support the hypothesis that the perceived sound depends on the eyes as well as the ears. Moreover, they would indicate cross-modal integration effects to be pervasive, intervening in all types of vocal articulation, indicating robust cross-modal integration of sight and sound.

## Experiment 1: estimating interval size

We reasoned that the visual influence of seeing a singer articulating an interval that is either ‘larger’ or ‘smaller than the one actually heard should be reflected systematically in judgments of interval size or the distance between the two consecutive tones. That is, when the seen interval belongs to an interval where the pitches are more ‘distant’ from each other, one should judge the interval as of larger size compared to the natural condition or original video. Conversely, when the seen interval belongs to an interval where the pitches are ‘closer’ to one other, one should judge the interval as of smaller size compared to the natural condition. Moreover, these stretching or compressive effects should vary proportionally to the tonal distance implied by the seen interval.

We tested 10 intervals, varying from a difference of one semitone (minor 2nd) to fourteen semitones (Major 9th). Each interval was shown 10 times, once with the congruently matching video (e.g., perfect 5th audio with perfect 5th video) and the other nine times with incongruent matches of videos from all the other intervals (e.g., perfect 5th audio with Major 3rd video, or with minor 9th, etc.). Each participant estimated, right after each video, ‘how distant apart the two tones sounded’ on a numerical scale from 1 to 14. Experiment 1A showed one female singer (S_1_) and Experiment 1B showed another female singer (S_2_). The participants were naïve to either the identity or range of interval distances used for both the A (N = 32) and B versions (N = 34). In addition, while viewing the videos, each participant’s gaze and pupil diameters were monitored with an infrared eye-tracker to obtain information about both the location of overt attention (gaze) and the intensity of attentional allocation (pupil size). These two oculomotor measurements allow us to assess, respectively: (a) the part of the singer’s face that captured attention the most (either lips or eyebrows); and (b) listeners’ arousal levels (using pupil size as proxy^27^) while listening to a specific audiovisual combination, reflecting the degree of excitement or the intensity of processing^28^ during audiovisual integration^29^.

### Results

The main dependent variable was the change in interval size estimates when the video showed facial movements belonging to either a larger musical interval or a smaller one, compared to the ‘same’ condition where the videos were the original ones. This was computed by subtracting from the Perceived Interval Distance (as indicated by the key press on the 14 steps scale) the real physical distance of the heard interval (also in 14 steps or distance between semitones). The average perceived distances of each participant was used as the dependent variable in a repeated-measure ANOVA, with Visual Interval (larger, same, smaller) as the within-subject factor and Singer (S1 and S2) as the between-subjects factor.

There was a strong main effect of Visual Interval, F(2,128) = 1279.6, p < 0.001, η^2^_**p**_ = 0.95, as well as a strong interaction with Singer, F(2,128) = 160.9, p < 0.001, η^2^_**p**_ = 0.72. The main effect of Singer was small and marginally significant, F(1,64) = 5.8, p < 0.02, η^2^_**p**_ = 0.08.

**Figure 1:**
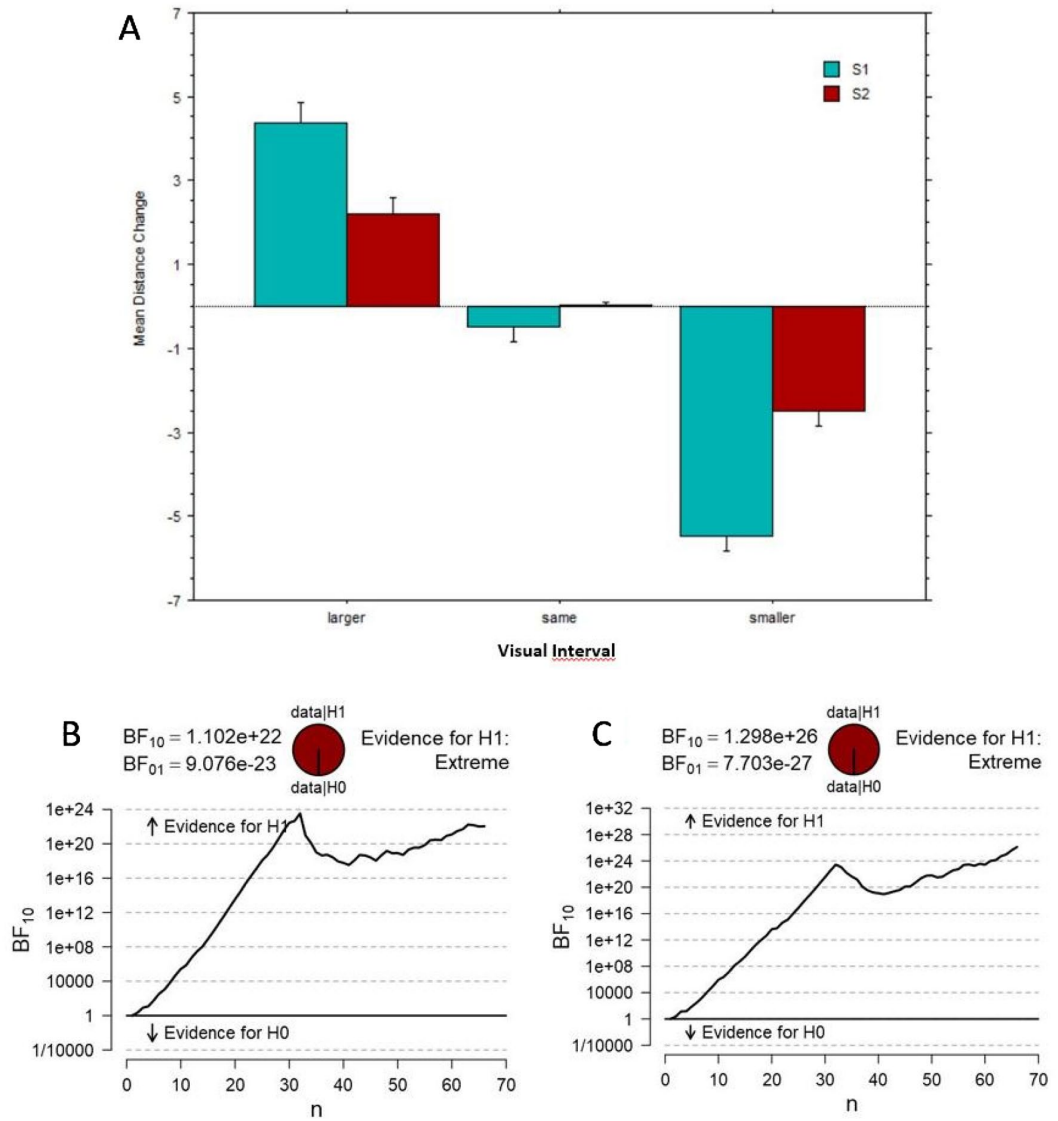
Experiment 1. (**A**) Bar graph of Mean Distance Change for visually larger, same, smaller intervals than the heard interval (split by Singers 1 and 2). Error bars are 95% confidence intervals. (**B**,**C**) Bayesian t-test sequential analyses of Larger Seen Interval versus Same intervals and Smaller Seen Interval versus Same Seen Interval, respectively.

As illustrated in Fig. 1A, there were clear expansion and compression effects on the perceived distance dependent on the visual interval displayed, essentially indicating the presence of a strong AV integration effect for both singers, though to a smaller extent for S2 than S1. Importantly, when the visual and auditory information were congruent (i.e., in the original videos), there was not significant change in size estimates from the actual number of semitones in the interval (Fig. 1A) in both Experiment A and B. In other words, participants did not show any significant expansion/compression in ‘same’ trials or the original AV clips and these remained remarkably close to zero or to the real semitone distance.

**Figure 2:**
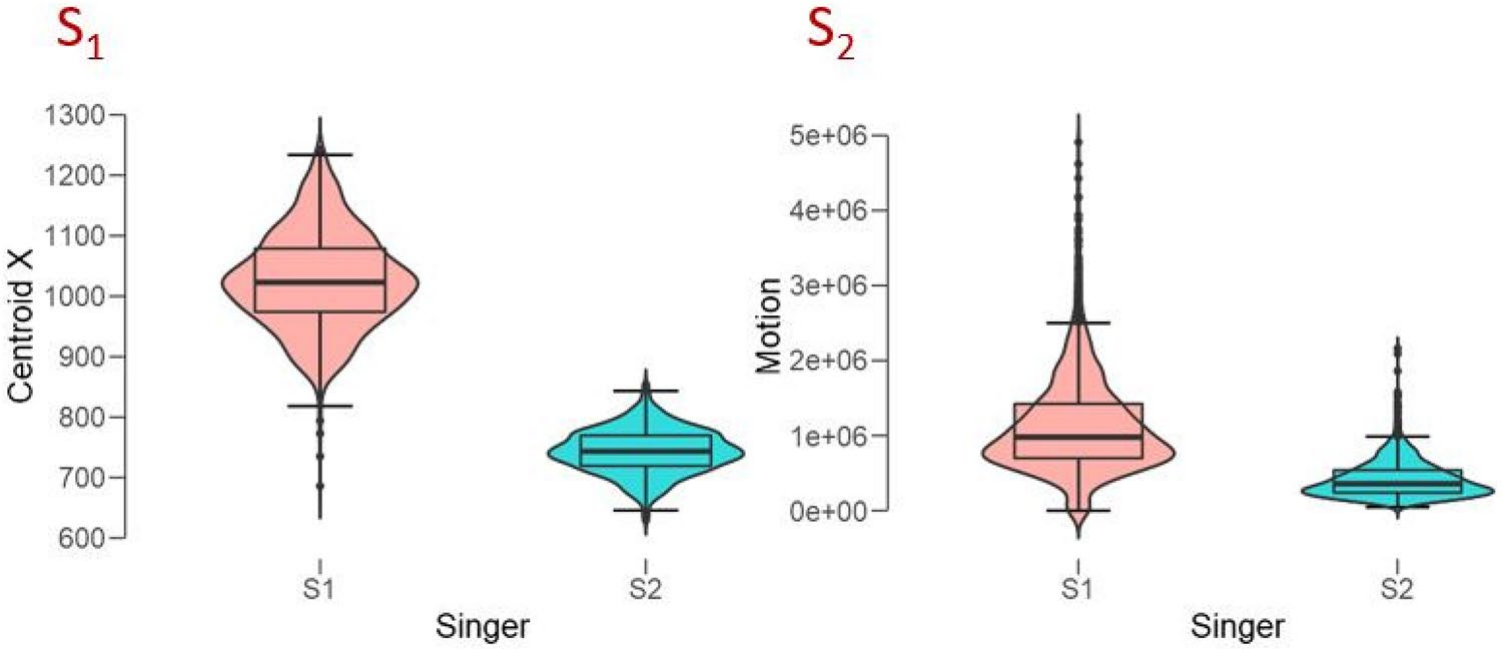
Experiment 1. Violin boxplots of motion captured from the videos of each singer (S_1_, S_2_) and for the two strongest differences (Left: centroid X; Right; global motion).

Nevertheless, the effect of incongruent trials was smaller for Singer 2 than for Singer 1, which could be due either to a difference among individuals in the two groups or to difference in the visual articulations between the two singers. The latter account is supported by a *motion capture* analysis from the videos of the two singers. There was more motion by Singer 1 than Singer 2, according to three captured parameters: Global Motion, t(2263) = 34.6, p < 0.001 (Cohen’s d = 1.46), Centroid X Motion, t(2263) = 110.8, p < 0.001 (Cohen’s d = 4.7), and Centroid Y Motion, t(2263) = 8.7, p < 0.001 (Cohen’s d = 0.4). Figure 2 illustrates the two strongest differences in violin boxplots.

**Figure 3:**
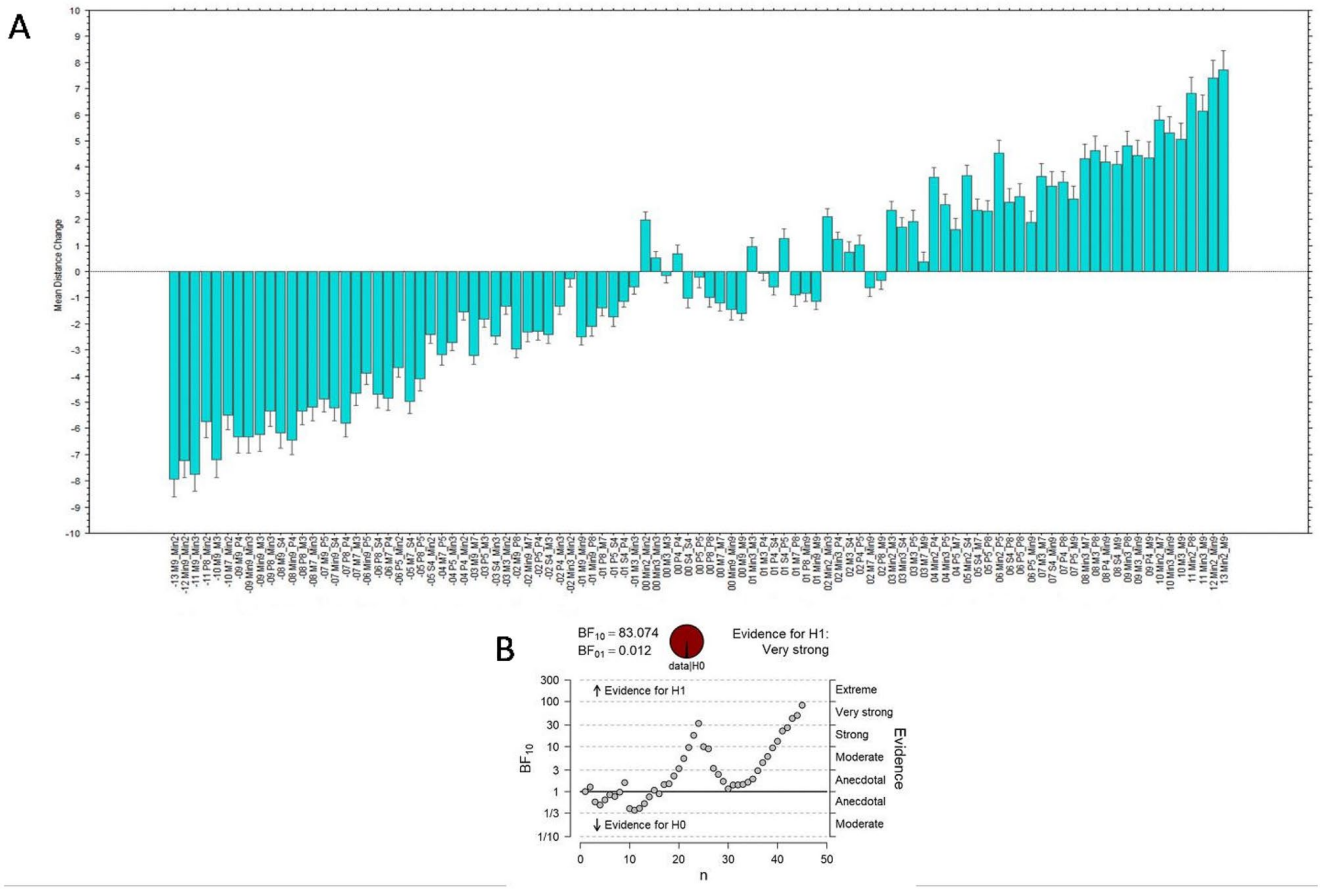
Experiment 1. (**A**) Bar graph of the perceived mean distance change, arranged by AV Semitone Distance (varying from − 13 to 13) and collapsed over Singers 1 and 2. Error bars are 95% confidence intervals. (**B**) Bayesian t-test sequential analysis of absolute mean distance change for Larger Seen Interval versus Smaller Seen Interval.

Moreover, the distance change in incongruent trials varied proportionally to the size of the seen intervals revealing clear compression and stretching effects when the seen interval was either smaller or larger than the heard interval (negative and positive values of Fig. 3A, showing the Mean Distance Change for semitone distances, varying from − 13 to + 13). Bayesian paired t-tests showed in two sequential analyses (for S1 and S2; Fig. 1B,C respectively) that there were no participants whose estimates supported the null hypothesis (H0) and the evidence for H1 was ‘extreme’ in both cases. In addition, considering incongruent trials only, we performed a paired t-test between the absolute (unsigned) mean distance changes for each AV pair and its reverse (e.g., ‘audio m2 & visual m9’ versus ‘audio m9 & visual m2’). This t-test revealed that the effect with visually smaller intervals (mean = 3.84; SD = 1.9) and the stretching effects with visually larger intervals (mean = 3.06; SD = 2.2) differed in magnitude, t(44) = 3.91, p = 0.0003. An additional Bayesian t-test sequential analysis (Fig. 3B) concluded for ‘extreme’ evidence for a difference in compression/stretching shifts and in favor of compression effects being stronger. We speculate that the facial movements associated with the visually smaller interval may be less compatible with larger auditory intervals, whereas the reverse (visually large movements and small auditory intervals) may be more likely to occur naturally.

A previous study using a similar interval-size estimation task with audiovisual mismatched clips^29^ found a negative relation between perceived shifts in interval size and the age of onset of musical training. Hence, we performed a linear regression analysis between the global score of the Goldsmith Musical Sophistication Index (GMSI) of each participant and the difference between average perceived distances for the larger and smaller visual intervals). The regression failed to reveal any relationship (r = 0.09). Another linear regression with the GSMI subscore of ‘years of musical training’ (range: 0–7 years) also failed to reveal a relationship (r = 0.06).

**Figure 4:**
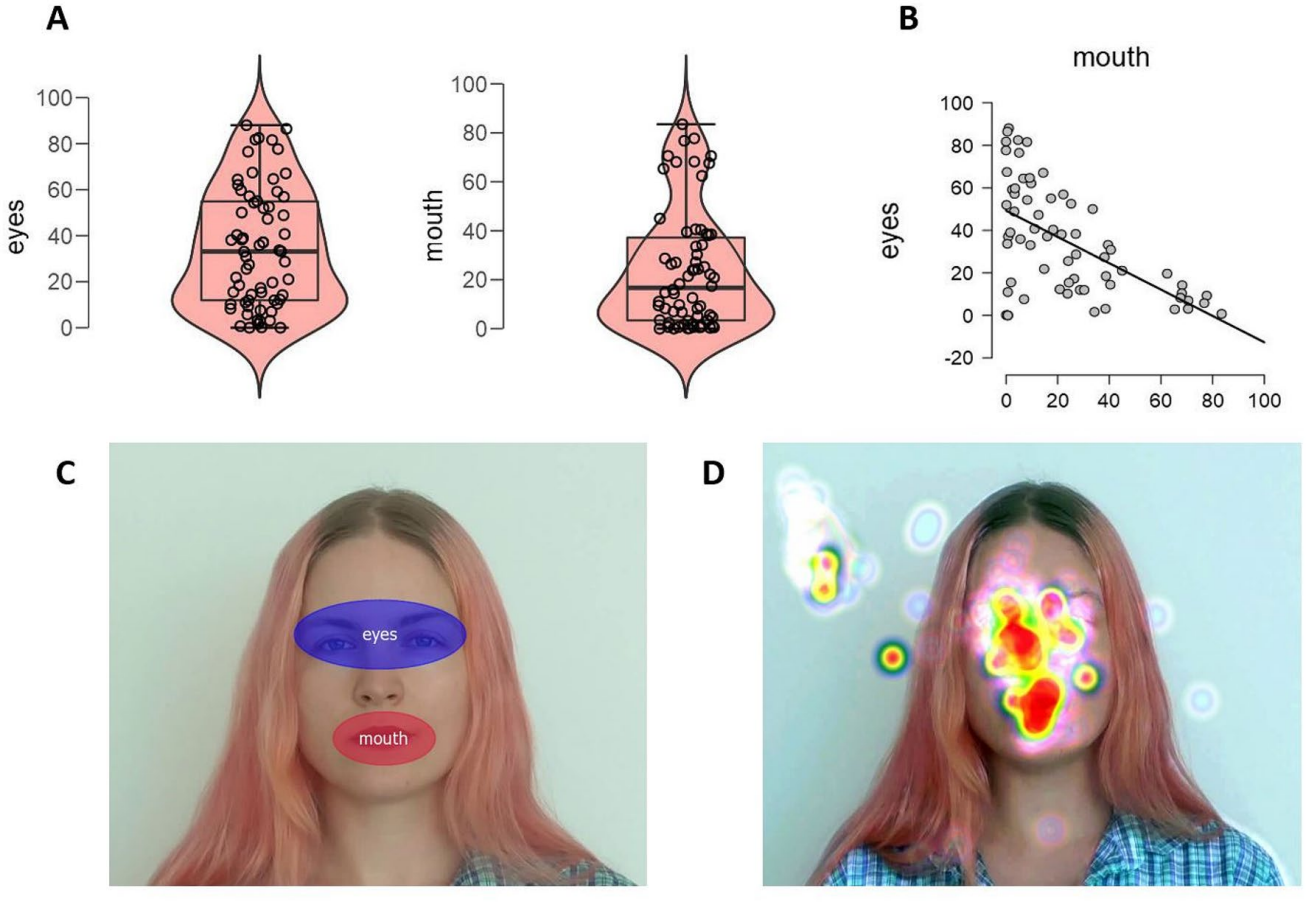
Experiment 1. (**A**) Violin plots of % Dwell Time on the AOI of eyes and mouth. (**B**) Scatterplot of % dwell time in the AOIs. (**C**) The AOIs superimposed to the video image. (**D**) Example heatmap while singing a P4.

To identify which part of the singer’s face was attended the most overtly, we computed the percent dwell time of gaze within areas of interest (AOI) corresponding to the most mobile parts of the face when singing: the mouth and eyes’ regions (including eyebrows). A repeated-measures ANOVA with Singer (S1 and S2) and AOI (eyes, mouth) revealed a highly significant effect of Singer, F(1, 64) = 17.2, p < 0.001, as well as a marginal effect of AOI, F(1, 64) = 3.97, p = 0.05, but no significant interaction between the two, F(1, 64) = 1.69, p = 0.20. Figure 4 illustrates the main results and the AOIs analyses. Regression analyses found a tendency to focus predominantly on one facial part (r = 0.33) but no significant relations between dwell times in AOIs and AV interval distance or Musicality (as global GSMI scores).

We also computed mean pupil averages of all participants in each trial. According to Kahneman’s^24^ psychophysiological model of ‘cognitive effort’, pupil diameters reflect general physiological arousal but, more specifically, they can reflect how intensively the cognitive system works at a specific point in time. In the present context, pupils could reveal how arousing or exciting stimuli are or the degree of processing required by AV integration. In the latter case, the pupil may dilate proportionally to degree of effort required by each episode of integration processing. Analyses revealed only a significant difference between the A and B blocks, t(59) = 6.56, p < 0.0001 (Singer 1: Mean = 4.55; SD = 0.12; Singer 2: Mean = 3.93; SD = 0.14.

## Experiment 2: detection of mismatching musical intervals

In the first experiment, participants made estimates of each musical interval based on a spatial metaphor: its size or distance between tone pairs. Although pitch is an acoustic phenomenon that has no real physical extension, for some musical instruments (e.g., strings, piano) the distance between two pitch is isomorphic to the spatial distance of fingers producing the sound (on the instrument’s keys or neck) or, in singing, the resonance center of the body. Most relevantly, head and facial movements in singers correlate to the size of the sung intervals^25^. Thus, using a metaphorical judgment of interval size may suggest, at least implicitly, matching it to the visible information (i.e., the extent of motion in the face). Thus, the results of experiments based on interval size estimates, although entirely consistent with the AV integration effect in the musical domain of pitch and intervals, remains suggestive but not decisive evidence for a change in the auditory perception of the intervals. In fact, in the classic McGurk effect, people can explicitly name hearing a different phoneme that was neither in the auditory nor in the visual input. However, for music, ordinary listeners do not have an explicit categorical recognition of intervals and cannot name the heard interval by stating its diatonic number or quality (e.g., a Major third).

Hence, in the second experiment, we sought converging and direct evidence for altered auditory perceptions by use of a different approach, based on a sample-to-match comparison task or, in other words, a judgment of acoustic similarity between two consecutively played intervals. That is, participants saw the same videos with Singer 1 used previously and, after a few seconds, heard an interval played on the piano. Half of the times, the two successive intervals were identical and, in the other half, the piano interval differed of a semitone from the one in the video. Participants chose a ‘yes’ or ‘no’ answer based on whether they heard the singer singing with precision the same interval than that played on the piano or whether they heard a different one, implying an error in the singer’s intonation. Note that, in Experiment 2, participants did not rate distance or use spatial metaphors as in the previous experiment; instead, they were simply required to judge the ‘likeness’ of sound.

Given the for a signal detection design is best to obtain unequivocal ‘hits’, ‘misses’, ‘correct rejections’ and ‘false alarms’ to compute the discrimination or sensitivity (d′) and criterion (C) values, we showed initially (A) only the normal AV clips and in another blocked condition (B), the same participants saw video-mismatches substituted in ‘same’ trials. We stress that the ‘same trials’ in B were identical in audio to the ‘same trials’ in A and differed only in (incongruent) visual information. All ‘different’ trials (i.e., with different audio intervals) were kept identical across blocks. Both the sung interval and the piano interval were equally-tempered, so that they mainly differed in timbre but in terms of pitch were identical (in same trials).

In other words, viewing the natural, unaltered, videos (A) first established a baseline measurement (d′) of each individual’s ability in matching the vocal and piano intervals. In the second block (B), in all trials where the auditory intervals of the voice and piano were identical, participants saw incongruent videos, where the singer sung an interval differing a semitone from the one heard simultaneously. If seeing a singer articulating a different interval does change what one hears, then participants should fail to perceive the identity of the vocal and piano intervals and judge them as different.

### Results

Since the experimental design allows two types of response (yes or no) and the two successive intervals can be either the same (Yes) or different (No), there are four possible outcomes in each block. Namely, a ‘hit’ when recognizing them as identical (yes, Yes); a ‘miss’ when hearing no identity (no, Yes); a false alarm (yes, No) when hearing different intervals as identical; a correctly rejection when spotting the difference between the two (no, No). Hence, these results can be submitted to a signal detection analysis, obtaining for each participant both sensitivity or *d′* and criterion or *C* measures^30^. By computing these separately for each block, we can test the prediction that sensitivity or *d′* decreases significantly in the second block, compared to the first, due to a change in the perceived vocal interval. Specifically, we expect a tendency to ‘miss’ hearing two identical intervals as the same, in the second (incongruent) block.

Even the baseline condition (Block A) proved to be challenging and six participants out of thirty-nine did not achieve a *d′* greater than zero, hence they were removed from further analyses. We computed individually the remaining participants’ responses to each Block with separate Signal Detection analyses^26^. These confirmed that *d′* scores were lower in Block B (mean *d′* = 0.717; SE = 0.125; mean *C* = − 0.112; SE = 0.071), compared to the Block A or baseline without dubbing (mean *d′* = 1.128; SE = 0.124; mean *C* = − 0.196; SE = 0. 066), t(32) = 4.6, p < 0.0001. In other words, when the Yes trials were incongruent so that, despite the interval sung and played on the piano were identical in audio, the videos showed singing an interval differing of a semitone, resulted in a decrease in perceptual sensitivity. Specifically, during the incongruent condition there was a significant decrease in sensitivity (d′) indicating a difficulty detecting that the voice and piano intervals were acoustically identical. Indeed, ‘miss’ responses increased from 25% in block A to 34% in block B, as confirmed by paired t-tests in ‘miss/hit’ responses between Blocks, t(32) = 3.3, p = 0.003. There were instead no differences in false alarms or correct rejections (1.6 < t < 1.7, 0.1 < p < 0.12) across blocks.

**Figure 5:**
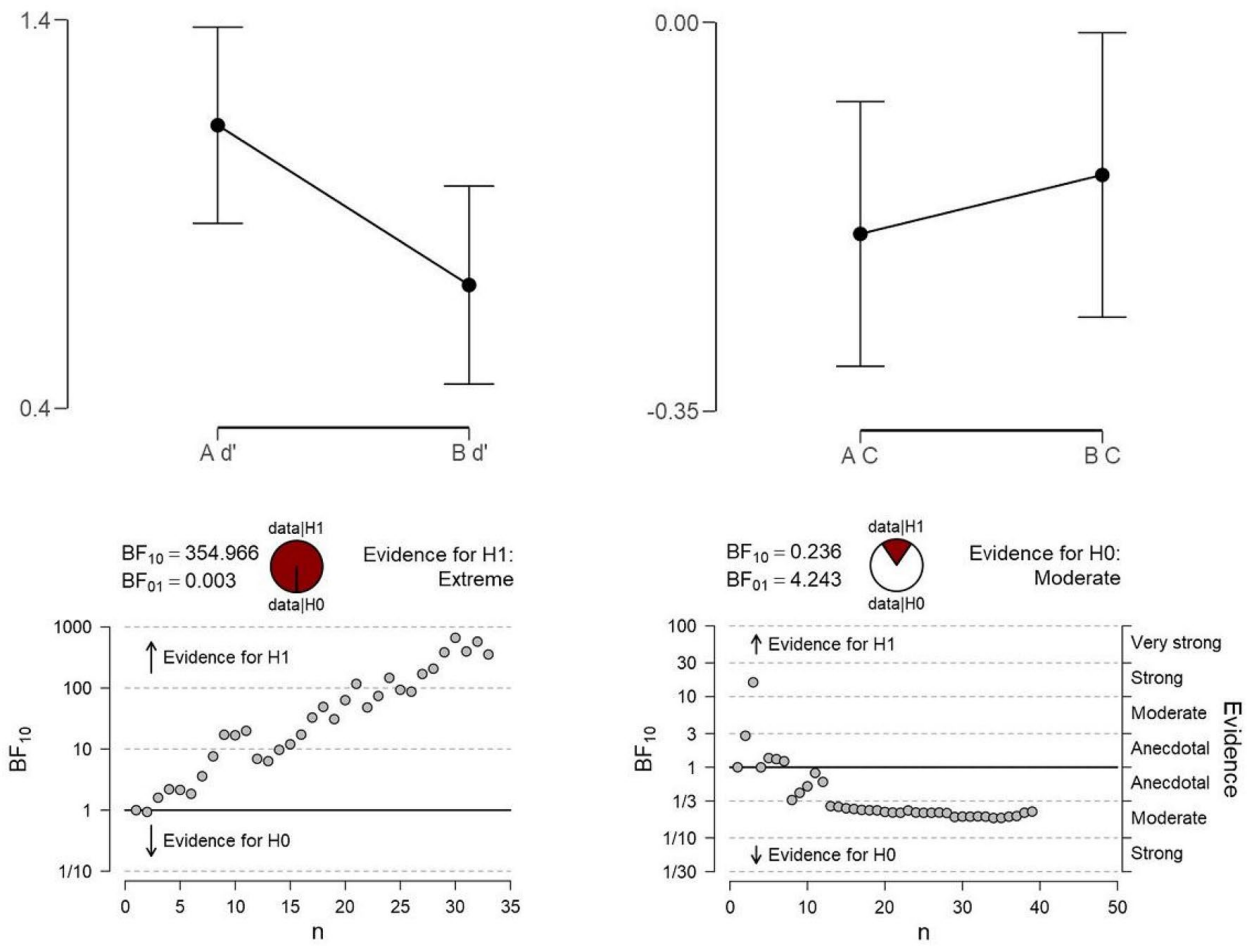
Experiment 2. Top: Line graphs (with confidence intervals) of the average d′ scores in block A and V (left) and of C in block A and B (right). Bottom: Bayesian t-test sequential analyses of sensitivity (d′) between Block A and B (i.e., original videos in A versus incongruent videos in B).

Figure 5 shows the mean d′ scores and mean C results as well as the Bayesian t-test sequential analyses of each block. Remarkably, no participant showed evidence for the null hypothesis (H0) for d′ and the analysis indicated the evidence in favor of H1 to be ‘extreme’. In contrast, there were no significant differences in Criterion or response bias, indicating that the introduction of the incongruent videos in block B did not result in changes of the participants’ decision criterion or bias.

## Discussion

Switching audio-videos of diatonic musical intervals in a same singer changed what music interval was heard, as reflected in clear expansion/compression effects of interval-size estimates of the musical intervals and in errors matching acoustically identical intervals that were played in succession. We found systematic and strong evidence that spontaneous musical gestures during singing (e.g., head movements or facial expressions like raised eyebrow or opening of the mouth) altered the heard interval’s relations, which lead to gross deviations in interval size estimates (in Experiment 1), and replicated and extended the findings of previous studies. Moreover, we found that repeated identical auditory intervals (in Experiment 2), where participants judged the likeness of intervals heard first on video and then on a piano yielded failed detections of identical musical intervals, despite they differed only by instrument. Hence, in both experiments, the parallel auditory/articulatory and visual/gestural ‘trajectories’ of audiovisual incongruent stimuli were bound into novel perceptions.

These effects occurred in nearly all individuals, indicating a strong susceptibility to cross-modal integration of sung tone intervals. Indeed, audio-visual integration of singing is highly expected, since every pitch and every interval has a certain degree of uncertainty. Previous psychophysical studies showed that shifts in heard musical pitch due to simultaneous sounds can approach a semitone^10^ and that, despite large individual differences (mainly due to musical training), the direction of shifts is the same for all listeners^31^.

Surprisingly, few studies have put to test the idea that audiovisual integration effects generalize to domains outside verbal behavior. This is particularly surprising considering music, since language and music share many perceptual and cognitive features^32–34^ and they may have co-evolved during the emergence of human cognition^35,36^. What one hears and sees in both speaking and singing often shares temporal frequency and amplitude features, despite each modality’s information is of a different nature^37^. Thus, perceiving music should strongly depend on combining vision to audition. Similarly to speech, generating music is linked to specific ‘articulations’, be these dependent on the vocal and facial apparatus for singing or on manual gestures for playing instruments.

The important question is whether vision can change essential elements of music like tones in the mind of the listener, just as consonants and vowels can with the McGurk effect. A previous study^23^ played the audio of a sung *tritone* or a *fifth* synchronized to videos displaying singing an *octave* above (+ 12 semitones difference) and participants estimated the interval’s size on a spatial scale; as expected, the incongruent or octave-matched audio–video yielded larger distance estimates or ‘stretching’ compared to the congruent audio–video. These findings were replicated in a subsequent study^24^ using five intervals in the audio and 13 ascending intervals in the videos. In the present study, we extended further the above results by using a fully cross-matched designed with 10 intervals, which helped revealing systematic expansion and compressing effects caused by the incongruent visual input. Remarkably, these distortion effects related to visual interval size in a clearly proportional manner (Fig. 3). Moreover, the cross-modal effects varied in strength according to the amount of motion displayed by different singers. Possibly, differences in the expressivity of the singers might be due to their different training durations and traditions (see “Methods”).

The integrative effects of vision and audition for singing stimuli in both experiments were extreme, as witnessed by the Bayesian sequential analyses (Figs. 1B,C, 5B,C). Remarkably, considering that for the McGurk effect on speech a percentage of individuals fails to show cross-modal fusion (e.g., about one third of the population^38,39^, none of our listeners showed evidence in favor of no cross-modal effect (i.e., Bayesian factors with BF_10_ smaller than 0.33^40^). Possibly, visual evidence may be more potent when ‘listening’ to singing than speaking, because of a wider range of auditory configurations of pitch to a same musical interval than of phonemes to a same auditory input. This may lead listeners to interpret the acoustical evidence for sung intervals in even more novel ways than with phonemes. However, the McGurk effect is similar with the /ba/ and /ga/ phonemes when these are either spoken or sung^41^.

We found that pupil diameters were larger on average for one singer than the other. Clearly, the more facially expressive singer (S_1_ according to the motion capture analysis (Fig. 2) aroused our participants more than the more restrained singer (S_2_). In general, vocal movements dilate observers’ pupils more compared to mere jaw and lip movements^42^ and for vocal melodies than when played on the piano^43^, possibly because vocal stimuli are both more arousing and information-rich, demanding more attention. We note that none of our participants explicitly reported detecting any perceptual conflict in the videos and, in fact, we did not find larger pupillary response to incongruent than congruent conditions. This also suggests that our listeners did not detect, at least consciously, the conflict or discrepancy between the auditory and visual information. This conclusion may be strengthened also by the gaze’s dwell times on the mobile parts of the face (eyes and mouth), which did not change with the strength of the AV integration effect.

Finally, scores in the musical sophistication questionnaire did not predict the effect of the incongruent visual information. This finding seems consistent with the idea that ‘common exposure’ to singers’ vocalizations is sufficient for generating the multimodal integration effects described here. In general, face-voice connections are learned early in life, since there is evidence for the McGurk effect already in the first half-year of life^44^ and before the emergence of speech. Considering how universal is across human populations to sing to babies (e.g., lullabies) while holding them close to the face^45^, it is possible that face-voice connections related to singing are also learned during infancy.

## Methods

### The participants

In Experiment 1A, we recruited 32 students as participants (24 females), ages ranging from 17 to 37. In Experiment 1B, we recruited another group of 34 students as participants (19 females), ages ranging from 16 to 35; in total 66 participants. In Experiment 2, we recruited 39 students as participants (14 females), ages ranging from 18 to 33. A power analysis using G*Power 3.1 was based on the a moderate effect size (Cohen’s *d* = 0.69) reported by Alsius and colleagues^46^ which estimated at least 19 participants, given α = 0.05, and power = 80%. Hence, the present number of participants exceeded the estimated sample size. Each participant was remunerated with a gift card of 200 NOK (about 19 Euro). The current project was ethically evaluated and approved (Ref. No. 3568281) by the IRB of the Department of Psychology, University of Oslo, and the research was performed in accordance with its guidelines and in accordance with the Declaration of Helsinki. We obtained informed consent from all participants.

### The singers

The two singers, S1 and S2 are both professional singers with several years of experience. Singer S1 was primarily trained in north Indian classical music (NICM) since the age of 3 and was later trained in western classical voice while learning operatic classical singing at conservatory level. Singer S2 was primarily trained in voice as a teenager and has taken part in ensembles, including modern classical musical ensembles. She currently sings in a band.

### The videos

We original recorded short audio–video clips of two singers, each singing 10 different musical intervals in the scale of C, varying from a difference of one semitone (minor 2nd) to fourteen semitones (Major 9th), i.e.: min2, min3, M3, S4, P4, P5, M7, P8, min9, M9. Subsequently, we separated the visual and audio components to combine them in 10 combinations of heard and seen intervals where nine were ‘incongruent’ or superimposed into novel and incongruent audiovisual intervals. Specifically, we recorded the original video recordings with a Panasonic HD video camera, placed on a tripod. Each singer’s face was centered in the camera’s sight and filmed against a white wall. Each recording consisted of two sequential tones, creating well-known musical intervals. Before each recording, a reference key of C4 (261.63 Hz) at a tempo of 120 BPM was played to the singer from a smart phone. Each video clip consists of each tone being sung for two beats, stopping at 4 beats. We instructed both singers to sing the two tones in this order, while maintaining the most neutral vocal and facial expression, without compromising vocal efficacy. We processed each of the 10 original files as audio files with *Logic Pro X* software and the *Flex* function to the frequency calculated by the just intonation tuning reference built into the software, so that all audio intervals in this study were ‘just-tempered.’ The pitch transition between the notes was smoothed, but not to a value exceeding 30% in order to preserve the original vocal transition characteristics.

Subsequently, we cross-dubbed the obtained 10 audio files and 10 video files without audio for each singer to create 100 files, matching each video interval with each audio interval using *Windows Video Editor* Software. The metronomic alignment of each stimuli ensured that there were no harsh jump-gaps between any notes. The resulting 100 stimulus video files were imported into a SMI Experiment Center script that controlled their presentation as well as the recording with the eye tracker.

### Motion capture from videos

We analyzed the singers’ motion from the videos using the Musical Gestures Toolbox for Matlab^47^. Motion analysis on the video files was based on MATLAB (MathWorks). We used the *mgmotion* class and the motion function, which can output, among several features, the quantity of motion *(qom)* and centroid of motion *(com)*. These we calculated by first converting the rgb image to greyscale. Then, a motiongram was generated using the following equation for two video frames: I *(x,y,t)*, I *(x,y,t* + *δt)*, the motion image at time t and, t + δt respectively is given by the equation ${I}_{mo}(t+{\varvec{\delta}}{\varvec{t}})\boldsymbol{ }=\boldsymbol{ }\left|{\varvec{I}}({\varvec{x}},{\varvec{y}},\mathrm{t}+\mathrm{\delta t})\boldsymbol{ }-\boldsymbol{ }\right.\left.{\varvec{I}}({\varvec{x}},{\varvec{y}},{\varvec{t}})\right|$** .** For an image I _*(x,y,t)*_ at a time t, the quantity of motion is computed by $QoM\left(t\right)= \sum_{x=1}^{m}\sum_{y=1}^{n}I (x,y,t)$. The centroid of motion was also computed from the motiongram. For an image with two motiongrams I_*gramx*_ and I_*gramy*_, the CoM was calculated as the fraction of summation over motiongrams in each axis of the video frame for each coordinate axis.

### Eye tracking

The eyes were tracked with an SMI R.E.D. infrared eye tracker at a sampling rate of 60 Hz to record oculomotor data (pupil diameters, gaze fixations) using an infrared light sensitive video camera. Instructions in English were given on a flat DELL LCD monitor with a screen resolution of 1680 × 1050, installed above the RED video camera. The distance from the participant’s eyes to the monitor and eye tracking device was set to 60 cm.

Experiment Center by SMI controlled the automated 4-point calibration at the beginning of the experiment. BeGaze (SMI) software was used to extract gaze dwell times within two identically sized areas of interest (AOI), corresponding to the mouth and the eyes’ regions (the latter including also the eyebrows). BeGaze allows adjusting dynamically the position of the AOIs to follow the underlying areas or face parts while they move (Fig. 4C,D; Nota Bene: We have consent to publish the image from the singer in an online open access publication). The datasets and video materials generated during and/or analyzed during the current study are available in the https://figshare.com/ repository (see Data Availability section, page 16).

### The pupils

Average pupil diameters in each fixation were extracted also with BeGaze (SMI) software, thus excluding artifacts due to blinks. Then, we obtained a grand pupil average in each trial by averaging the pupil diameters of the left eye. Six participants had missing pupil data and were excluded from the group analyses (N = 60).

### Signal detection

We selected videos from the set of Experiment 1A. 4 s, heard an interval played on the piano. Half of the times, the two successive intervals were identical and, in the other half, the piano interval differed of a semitone from the one in the video. After the piano interval, participants clicked on the ‘yes’ or ‘no’ options on screen presented via the questionnaire function of SMI Experiment Center. In the first block, we used only the natural, unaltered, videos as a baseline measurement, while in the second block, in ‘same’ (yes) trials, participants always saw an incongruent video, with the visual interval differing a semitone from the acoustic one.

## Data Availability

The datasets and video materials generated during and/or analyzed during the current study are available in the https://figshare.com/ repository: Experiment 1A data (https://figshare.com/s/55bfab0bf3564aa536a2), Experiment 1A videos (https://figshare.com/s/769b7c41d57261511acb), Experiment 1B data (https://figshare.com/s/c616b784a6036aa66cf0), Experiment 1B videos (https://figshare.com/s/d0b5d6f7acbdd4b15464), Experiment 2 Block A data (https://figshare.com/s/4c161d7bb9edd2774783), Experiment 2 Block B data (https://figshare.com/s/aa223789732c8fb62fe5), Experiment 2 Piano sound files (https://figshare.com/s/7b0668c9f7da247c2951).
